# Pyruvate kinase M2 is elevated in systemic sclerosis and plays a role in its pathogenesis

**DOI:** 10.3389/fimmu.2026.1827119

**Published:** 2026-07-23

**Authors:** Thomas Steadman, Steven O’Reilly

**Affiliations:** Biosciences Department, Durham University, Durham, United Kingdom

**Keywords:** epigenetics, fibroblast, fibrosis, lactylation, metabolism, PKM2, systemic sclerosis

## Abstract

**Background:**

Systemic sclerosis is an autoimmune fibrotic skin disease characterised by immune activation and fibrosis. Fibroblasts are at the core of this fibrotic process, differentiating into effector myofibroblasts; however, the drivers of this process remain obscure. Recently, metabolic changes in cells in fibrotic diseases have been uncovered, with changes in the TCA cycle and glycolysis being observed.

**Objective:**

The objective of this work was to elucidate the role of the glycolytic enzyme pyruvate kinase M2 (PKM2) in systemic sclerosis.

**Methods:**

Serum from early diffuse systemic sclerosis (SSc) patients and healthy controls (HC) was collected for PKM2 analysis by ELISA. Fibroblasts were isolated from HC and SSc biopsies and treated with transforming growth factor-beta 1 (TGF-β1) to assess PKM2 expression. PKM2 was pharmacologically modulated, and collagen and Extracellular Matrix (ECM) regulators were evaluated. Metabolic activity was assessed using Seahorse assays, and lactate transport was chemically inhibited. Chromatin immunoprecipitation was performed using a histone lactylation-specific or isotype antibody, and lactate or acetate supplementation experiments were conducted.

**Results:**

We found significantly elevated circulating and fibroblast PKM2 in SSc patients. Fibroblast PKM2 could be induced in healthy fibroblasts by TGF-β1 exposure. Furthermore, PKM2 drives a metabolic shift to glycolysis that can be blocked by forced tetramerisation of PKM2 from its dimeric form; this resulted in reduced ECM and matrix regulators. Mechanistically, PKM2-mediated glycolysis results in elevated lactate, which drives collagen via epigenetic regulation by histone H3K18 lactylation. Blockade of PKM2 dimerisation reduced Histone H3 at Lysine 18 (H3K18) lactylation at the collagen promoter, which could be restored with lactate but not acetate. We further demonstrated that lactate-induced collagen is partially mediated by HIF-1α.

**Conclusion:**

PKM2 drives activation of fibroblasts in SSc via metabolic changes such as glycolysis. Taking advantage of PKM2 tetramerisation or blockade of lactate generation could be a possible therapeutic option in a disease with few treatment options.

## Introduction

Systemic sclerosis (SSc) is an autoimmune connective tissue disease that has three cardinal features: inflammation, vascular rarefaction, and fibrosis (1). The disease is characterised by inflammation and fibrosis, which are mediated by the activation of quiescent fibroblasts into myofibroblasts, which deposit excessive extracellular matrix, have increased contractility, and are resistant to apoptosis (2). The fibrosis primarily affects the skin and can affect internal organs such as the lung and heart (3). Of all the inflammatory rheumatic diseases, SSc has the highest all-cause mortality, and although advances have been made in the last 10 years, no therapeutic modality exists that modifies the fibrotic element of the disease.

The critical cell type in fibrosis in SSc is the myofibroblast, which arises from a resident quiescent fibroblast and acquires a highly contractile phenotype and produces excessive ECM, including collagen and fibronectin. A classic activator of fibroblasts is the profibrotic cytokine transforming growth factor-beta 1 (TGF-β1), which is a prototypical fibrosis-inducing cytokine. The precise mechanism that governs myofibroblast generation remains unclear. Recently, the role of metabolism and glycolysis has been implicated in fibrosis (4, 5). Ding et al. demonstrated that inhibiting glycolysis reduced myofibroblast accumulation in renal fibrosis *in vivo* (5). We demonstrated in SSc fibroblasts that glycolysis and glutaminolysis are important events underpinning the generation of myofibroblasts (6).

Pyruvate kinase M2 (PKM2) is the rate-limiting enzyme in glycolysis, converting phosphoenolpyruvate (PEP) to pyruvate (7). There are four different isoforms of PK in mammals, but PKM1 and PKM2 are widely expressed in many different cell types and are generated by mutually exclusive alternative splicing (8). PKM2 can be found in either a dimeric or tetrameric form and is subject to allosteric regulation. The PKM2 dimer has low pyruvate kinase activity and promotes aerobic glycolysis (9), can translocate to the nucleus to initiate gene transcription through multiple mechanisms (10, 11), and, in cancer, may include stabilisation of hypoxia-inducible factor-1 α (HIF-1α). The tetrameric form of PKM2 has higher enzymatic activity, favouring the production of pyruvate and ATP. Thus, the different allosteric forms of PKM2 can regulate different signalling pathways via metabolic and nonmetabolic mechanisms. PKM2 expression is significantly implicated in cancer development, with high expression in tumours (12). This is associated with glycolysis even in the presence of adequate oxygen, a process known as the “Warburg effect”. The effect of PKM2 may be due to its positive effect on HIF-1α (13), which is also ERK1/2-dependent (14). Recently, PKM2 noncanonical signalling has been associated with fibrosis, including liver fibrosis, in which it modulates the macrophage phenotype, promoting fibrosis (15). PKM2 has been demonstrated to be key in kidney fibrosis development (5, 16), and most recently, it was shown to play a significant role in pulmonary fibrosis (17, 18). Given that hypoxia is prevalent in SSc (19) and that we have also demonstrated metabolic perturbations in SSc (6), we sought to examine the role of PKM2 in SSc.

## Patients and methods

Thirty patients with early diffuse SSc were involved in the study; this was a single-centre study. All patients fulfilled the American College of Rheumatology (ACR) criteria for a diagnosis of systemic sclerosis and were diagnosed as having the diffuse subset by a rheumatologist. Full informed consent was obtained from the patients, who were seen at Newcastle Hospital Trust, UK. Patients were defined as having early diffuse SSc, with disease duration of < 2 years since the first non-Raynaud’s symptom. The study received full ethical approval from the local research ethics committee (REC) (Approval No. REC/13/NE/0089) and followed the Declaration of Helsinki guidelines. Healthy controls were age- and gender-matched (*n* = 30). A total of 15 ml of blood was drawn from each donor’s arm, and serum was isolated by centrifugation at 2,000×*g* for 15 min. Sera were frozen at − 80 °C until use.

In five patients, biopsies were taken at baseline and frozen immediately, and additional biopsies were taken 12 months posttreatment with mycophenolate mofetil (MMF) < 2 g/day. Serum was also taken, and enzyme-linked immunosorbent assay (ELISA) was performed as detailed below.

### ELISA for serum PKM2 levels

Serum was used from 30 patients with early diffuse SSc and 30 healthy controls. ELISA was performed using the PKM2 sandwich ELISA (Cloud-Clone Corp, China) using a 96-well plate and read using a plate reader. A follow-up analysis of five patients treated with MMF was also performed using this ELISA after 12 months.

### Cell isolation

A 4-mm punch biopsy was taken from the affected fibrotic area of skin on the arm under local anaesthesia. SSc dermal fibroblasts were isolated and cultured in standard fibroblast culture medium consisting of Dulbecco’s modified Eagle’s medium (DMEM) supplemented with 10% heat-inactivated foetal calf serum (FCS), 2 mM l-glutamine, and 100 U/ml penicillin/streptomycin (Sigma-Aldrich, UK) at 37°C in an atmosphere containing 5% CO_2_. Fibroblasts were isolated by separating the epidermis from the dermis and mincing the tissue with a sterile scalpel in a six-well plate. Cells were cultured and used between passages 1 and 5 in standard tissue culture medium. Cells from healthy controls were isolated as described above from individuals undergoing fat reduction surgery (abdominal tissue) and were gender-matched. The fibroblast cultures were all used between passages 1 and 5.

### ELISA for TGF-β, TIMP-1, and MMP-1

Cell culture medium was removed from the SSc fibroblasts after stimulation with Dimethylsulphoxide (DMSO) vehicle or TEPP-46, and ELISA was performed using the TGF-β1 Quantikine (R&D Systems, UK), Tissue Inhibitor of Matrix metalloproteaniase-1 (TIMP-1) Quantikine (R&D Systems, UK), and MMP-1 (R&D Systems, UK) kits, all of which were quantified in triplicate using 96-well plates according to the manufacturer’s instructions.

### TGF-β1 treatment and inhibition

Healthy dermal fibroblasts were cultured between passages 1 and 5 and treated with either control DMEM medium or TGF-β1 (10 ng/ml; R&D Systems, UK) for 20 h. After 20 h, RNA was isolated, and qPCR, as described below, was performed. In some experiments, cells were treated with SB431542 (10 μM) or DMSO vehicle control (0.1% vol) for 1 h. After 1 h of pretreatment, TGF-β1 (10 ng/ml; R&D Systems) was added, and after a further 20 h, RNA was isolated from the cells, or after 24 h, collagen protein quantification was undertaken. The concentration of 10 μM was chosen because this has previously been used in primary dermal fibroblasts (20). To inhibit the downstream transcription factor Smad3, the specific inhibitor SIS3 at 30 μM was used (21). Healthy dermal fibroblasts were cultured with SIS3 (30 μM) or vehicle control for 1 h before the addition of TGF-β1 (10 ng/ml) for 20 h. This concentration was chosen because it was titrated using a Smad reporter cell line.

### Conditioned medium transfer experiments

Healthy control and SSc dermal fibroblasts were cultured and medium collected after 3 days in culture (*n* = 5 separate donors). This conditioned medium was then added to healthy dermal fibroblasts in 24-well plates after the cells were left to settle for 24 h, and the medium removed and cells washed twice with sterile phosphate-buffered saline (PBS) before the Conditioned media (CM) was added. In some experiments, SSc CM was supplemented with the 1d11 antibody (1 μg/ml), or a matched-concentration IgG control Ab was added (both Thermo Fisher, UK).

### Polyacrylamide hydrogels

Hydrogels were prepared using 40% acrylamide and 2% *bis*-acrylamide (Bio-Rad, UK). Acrylamide solution for soft (0.6 kPa) and stiff (6 kPa) gels was applied to oxygen plasma-activated sterile 25-mm coverslips. A coverslip was then placed on top of the gel. After polymerisation of the gels, the coverslip was removed, and sulpho-SANPAH (1 mg/ml) was applied and UV irradiated (365 nM), washed in sterile PBS, and then incubated with type I collagen (50 μg/ml) at 4 °C overnight. After overnight incubation, the hydrogels were washed once in sterile PBS, and healthy dermal fibroblasts were placed upon the hydrogels. In some experiments, the cell-laden stiff hydrogel received the Yes-associated protein (YAP) inhibitor verteporfin (10 μM; Tocris, USA) or DMSO vehicle control in DMEM medium. After 48 h of culture, the cells were removed, and qPCR for PKM2 was performed with 18S as the housekeeping gene. Data are shown as fold change relative to soft hydrogel (0.6 kPa).

### PKM2 tetramerisation in SSc cell fibroblasts

SSc dermal fibroblasts were cultured in 24-well plates, then exposed to DMSO vehicle control (0.1% vol) or the PKM2 modulator TEPP-46 (40 μM) (Medchem Express, UK), after which downstream analysis occurred. This concentration was chosen based on previous studies.

SSc dermal fibroblasts were cultured as above and treated with vehicle control or the PKM2 activator DASA-58 (10 μM), after which collagen protein secretion was measured using the Sircoll assay after 30 h of exposure.

### Syrosingopine treatment in healthy dermal fibroblasts

SSc dermal fibroblasts were cultured in 24-well plates, then exposed to DMSO vehicle control or the lactate-importer monocarboxylate transporter-1 (MCT-1) inhibitor syrosingopine (10 μM; Sigma-Aldrich) for 1 h before the addition of TGF-β1 (10 ng/ml) for both treatments for an additional 48 h. After the 48-h exposure, collagen was quantified and TIMP-1 by ELISA, alongside connective tissue growth factor (CTGF) and interleukin (IL)-6 messenger RNA (mRNA) expression.

### Sircoll Assay for collagen quantification

Secreted collagen was quantified by the Sircoll Assay in culture medium (Biocolor, Belfast, UK) as previously described (22).

### qRT-PCR

Quantitative reverse transcription polymerase chain reaction (qRT-PCR) was performed after RNA isolation and reverse transcription using Nanoscript 2 reverse transcriptase (Primer Design, USA) with 1 μg of RNA input. cDNA was amplified using SYBR Green Taq Ready (Sigma-Aldrich, UK) and primers specific for human PKM2 (Forward: 5′-CGA ATG GCT CAT TAA ATC AGT TAT GG-3′; Reverse: 5′-TAT TAG CTC TAG AAT TAC CAC AGT TAT CC-3′), connective tissue growth factor (Forward: 5′-CTCGCGGCTTACCGACTG-3′; Reverse: 5′-GCACTTGAACTCCACCGG-3′), ACTA2, IL-6 (Forward: 5′-ACTCACCTCTTCAGAACGAATTG-3′; Reverse: CCATCTTTGGAAGGTTCAGGTTG-3′), and IL-1β (Forward: 5′-AGCTACGAATCTCCGACCAC-3′; Reverse: 5′-CGTTATCCCATGTGTCGAAGAA-3′) using the 7500 RT-PCR system (Applied Biosciences, UK). All data were normalised using 18S expression as the internal control and analysed using the ΔΔCt method to calculate fold change relative to control. A no-template control was included on each plate.

### Chromatin immunoprecipitation

Healthy dermal fibroblasts were cultured in 10 cm dishes and treated either with standard media or media supplemented with Na-lactate (20 mM). In some experiments, cells were treated with C464 with lactate or DMSO (0.1%) vehicle. After 20 h, the cells were fixed with formaldehyde (37%) and quenched with glycine (0.125 M). Following lysis with SDS buffer (11% SDS, 10mM EDTA, 50 mM Tris-HCl, pH 8.1) containing protease inhibitors, lysates were sonicated. Then samples were precleared using protein G-agarose beads and subsequently incubated with anti-H3K18La (1:80, PTM Biolabs, USA) or IgG antibody at 4 °C overnight, followed by precipitation with protein G-agarose beads. After elution of the immunoprecipitated complex, crosslinking was reversed, and proteins were digested with proteinase K and RNase. Chromatin from the proximal promoter region of collagen1A1 was detected by quantitative PCR using SYBR Green with primers (Forward: 5′-TGCTGTTCTTGGGGGACTAC-3′; Reverse: 5′-GCCATAGAGGGGTGTTCTCA-3′). Data are presented as fold enrichment relative to the IgG control. Experiments were performed using fibroblasts from four healthy donors or SSc cells. In some experiments, cells were additionally exposed to C464 (1.2 μM) or DMSO vehicle for 1 h prior to lactate treatment, followed by chromatin immunoprecipitation (ChIP) analysis.

### Cell viability assay

After treatment with either vehicle or TEPP-46 (40 μM) or DASA-58, cell viability was quantified using the CyQUANT MTT assay (Thermo Fisher, UK) and measuring the absorbance at 570 nm. Data were compared to vehicle and shown as percentage viability, with vehicle set at 100%.

### 3-OBA exposure

Fibroblasts were cultured in 24-well plates and exposed to the G protein-coupled receptor 81 (GPR81) antagonist 3-OBA (500 nM) for 1 h prior to treatment with Na-lactate (20 mM) or lactate alone. Collagen was then quantified by the Sicoll assay.

### Lactate treatment VEGF measurement (qRT-PCR)

Cells were treated with Na-lactate (20 mM) for 24 h, after which they were lysed, and RNA was harvested for qPCR measurement analysis of Vascular Endothelial Growth Factor (VEGF) expression. As described in the qPCR methods, data were normalised to the 18S housekeeping gene and expressed as fold change relative to untreated cells.

### HIF-1α inhibition experiments

Dermal fibroblasts were cultured and pretreated for 1 h with the specific HIF-α inhibitor PX-478 (10 µM; Sigma-Aldrich, UK), the HIF-1α inhibitor 3-OBA (500 nM), or DMSO vehicle control prior to the addition of Na-lactate or untreated. After 24 h, collagen levels were quantified using the Sircol assay.

### Tofacitinib treatment

Dermal fibroblasts were cultured and pretreated for 1 h with the JAK inhibitor tofacitinib (100 nM; Merck, USA), followed by Na-lactate or no treatment. As a positive control, cells were exposed to recombinant IL-6 (20 ng/ml) and soluble IL-6 receptor (25 ng/ml) (both from R&D Systems, UK). IL-6 trans-signalling is needed because dermal fibroblasts do not express the IL-6 receptor. In additional experiments, cells received IL-6 and sIL-6 receptor together with tofacitinib (100 nM) or DMSO vehicle control treatment. After 24 h of treatment, collagen levels were quantified using the Sircol assay.

### STAT3 luciferase reporter

Healthy dermal fibroblasts were transfected with the Signal Transducer and Activator of Transcription 3 (STAT3) luciferase reporter Cignal (100 ng/μl; Qiagen, UK) using Lipofectamine transfection reagent. Twenty-four hours posttransfection, cells were exposed to lactate or IL-6 (20 ng/ml) and sIL-6R (25 ng/ml) for 12 h. Luciferase was measured using a luminometer. Transfection efficacy was normalised to Renilla luciferase activity, and data were expressed as fold change relative to luciferase-transfected but untreated cells.

### ECAR measurement

Glycolysis was measured using a Seahorse XFp analyser. Healthy dermal fibroblasts were seeded at 0.1 × 10^6^ cells/well in a Seahorse XFe 24-well plate and allowed to adhere for 3 h before treatment with either vehicle control or the PKM2 activator TEPP-46, followed by TGF-β1 protein (10 ng/ml) for 12 h. On the day of the assay, cells were switched to Seahorse XF Base DMEM (pH 7.4; Agilent, UK) supplemented with 2 mmol/L glutamine. Glycolytic parameters were determined by measuring the extracellular acidification rate (ECAR) in response to sequential additions of glucose (10 mmol/L), oligomycin (1 µmol/L), and 2‐DG (50 mmol/L) (all from Sigma). Results were quantified as the change in pH/minute, normalised to total protein contents (μg).

### Lactate quantification

Lactate levels in dermal fibroblasts were quantified using a commercially available lactate assay kit (Abcam, UK). Cells were seeded into a six-well plate and then exposed to vehicle or TEPP-46 (40 μM) for the indicated duration.

Lactate concentrations were quantified using paired skin biopsies collected from affected areas at baseline and 12 months posttreatment. After freezing, tissue samples were weighed and homogenised in 50 mM Tris buffer (pH 7.5). Homogenates were mechanically processed, inactivation buffer was added, and subsequent steps were performed according to the manufacturer’s instructions. Data are expressed as millimoles per milligram wet weight.

### Statistical analysis

Comparisons between two groups were performed using Student’s *t*-test. For multiple group comparisons, one-way analysis of variance (ANOVA) with Bonferroni’s or Tukey’s *post hoc* correction was applied. Two-way ANOVA followed by Tukey’s correction was used where appropriate. Correlation analyses were conducted using two-tailed Pearson’s correlation between serum PKM2 values and the Rodnan Skin Score (mRSS); additional subgroup analyses were performed in patients treated with MMF for 12 months, assessing correlations between ΔPKM2and ΔmRSS, as well as between ΔPKM2 levels and Δlactate. *p-*values < 0.05 were considered statistically significant. Data are presented as mean ± SEM. All analyses were performed using Graphpad Prism software (version 10.6; USA).

## Results

Thirty patients with early diffuse SSc and 30 healthy controls were included in the study. **Table 1** provides an overview of patient demographics. Of the SSc cohort, 27 were women and three were men, with a mean age of 51.4 years (SD: 7.9; range: 35–66 years). None of the patients were receiving treatment at the time of inclusion. Disease was defined as early when the duration from the onset of the first non-Raynaud’s symptom was ≤ 2 years.

**Table 1:** Clinical characteristics of SSc patients.

Patient number	Age (years)	Sex	Autoantibodies	mRSSa	Treatment	ILD	DLCO%
Patient_1	48	F	Scl-70	9	None	N	85
Patient_2	54	F	Scl-70	16	None	N	82
Patient_3	51	F	RNA-polIII	10	None	N	89
Patient_4	39	F	Scl-70	11	None	N	73
Patient_5	52	F	Scl-70	12	None	N	79
Patient_6	41	M	Scl-70	10	None	N	87
Patient_7	42	F	Scl-70	21	None	N	91
Patient_8	57	M	Scl-70	14	None	N	75
Patient_9	35	F	Scl-70	19	None	N	82
Patient_10	47	F	Scl-70	11	None	N	76
Patient_11	61	F	Scl-70	15	None	N	74
Patient_12	37	F	Scl-70	18	None	N	78
Patient_13	66	F	Scl-70	16	None	Y	60
Patient_14	49	F	Scl-70	14	None	Y	57
Patient_15	55	F	RNA-polIII	17	None	Y	52
Patient_16	55	F	Scl-70	21	None	Y	50
Patient_17	48	F	Scl-70	22	None	Y	54
Patient_18	46	F	Scl-70	19	None	N	88
Patient_19	59	F	Scl-70	17	None	N	81
Patient 20	63	F	Scl-70	20	None	N	90
Patient 21	59	F	Scl-70	17	None	N	91
Patient_22	48	F	Scl-70	12	None	N	95
Patient_23	55	F	RNA-polIII	27	None	N	90
Patient_24	61	F	Scl-70	12	None	N	83
Patient_25	58	F	Scl-70	14	None	N	75
Patient_26	46	F	Scl-70	6	None	N	90
Patient_27	53	F	RNA-polIII	38	None	Y	60
Patient 28	47	F	Scl-70	12	None	N	88
Patient 29	59	M	Scl-70	18	None	N	75
Patient 30	51	F	Scl-70	12	None	N	78

^a^ Modified Rodnan Skin Score, maximum 0–51.

### PKM2 is elevated in systemic sclerosis

We sought to determine extracellular PKM2 levels in patients with SSc using ELISA. Circulating PKM2 was significantly elevated in early diffuse SSc compared with healthy controls (mean: 1.9 ng/ml, range: 0.1–4 vs. 0.28 ng/ml, range: 0–1.0; *p* ≤ 0.0001, Student’s *t*-test; *n* = 30).

**Figure 1:**
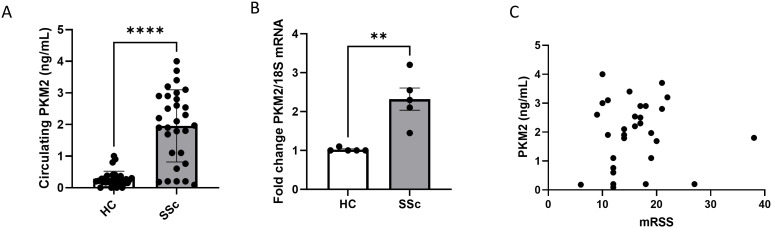
PKM2 is elevated in SSc. **(A)** Circulating serum PKM2 was measured by ELISA in healthy control or SSc serum from 30 donors (^****^p ≤ 0.0001, Student’s *t*-test; *n* = 30). Data are from individual donors, with the mean and SEM shown. **(B)** Isolated HC or SSc dermal fibroblasts were analysed for PKM2 gene expression by qPCR. Data are normalised to the 18S gene and presented as fold change compared with HC (^**^p = 0.019, Student’s *t*-test; *n* = 5). **(C)** No correlation was observed between serum PKM2 and mRSS (*r* = 0.051; 95% CI = − 0.3151 to 0.4038; *p* = 0.79, Pearson’s correlation; *n* = 30).

As circulating PKM2 levels were elevated in patients with SSc, we next examined the PKM2 gene expression in dermal fibroblasts isolated from the forearm of five SSc patients and compared them with HC fibroblasts. PKM2 gene expression was significantly increased in SSc fibroblasts (1.0 vs. 2.4-fold change; *p* = 0.0019, Student’s *t*-test; *n* = 5) (**Figure 1B**). No correlation was observed between serum PKM2 and the modified mRSS (*r* = 0.051, *R*^2^ = 0.0026; *p* = 0.79, Pearson’s correlation) (**Figure 1C**). Patients with SSc-associated ILD exhibited higher serum PKM2 levels compared with those without ILD.

### TGF-β1 regulates PKM2, and fibroblasts from patients with SSc secrete PKM2-modulating factors

**Figure 2:**
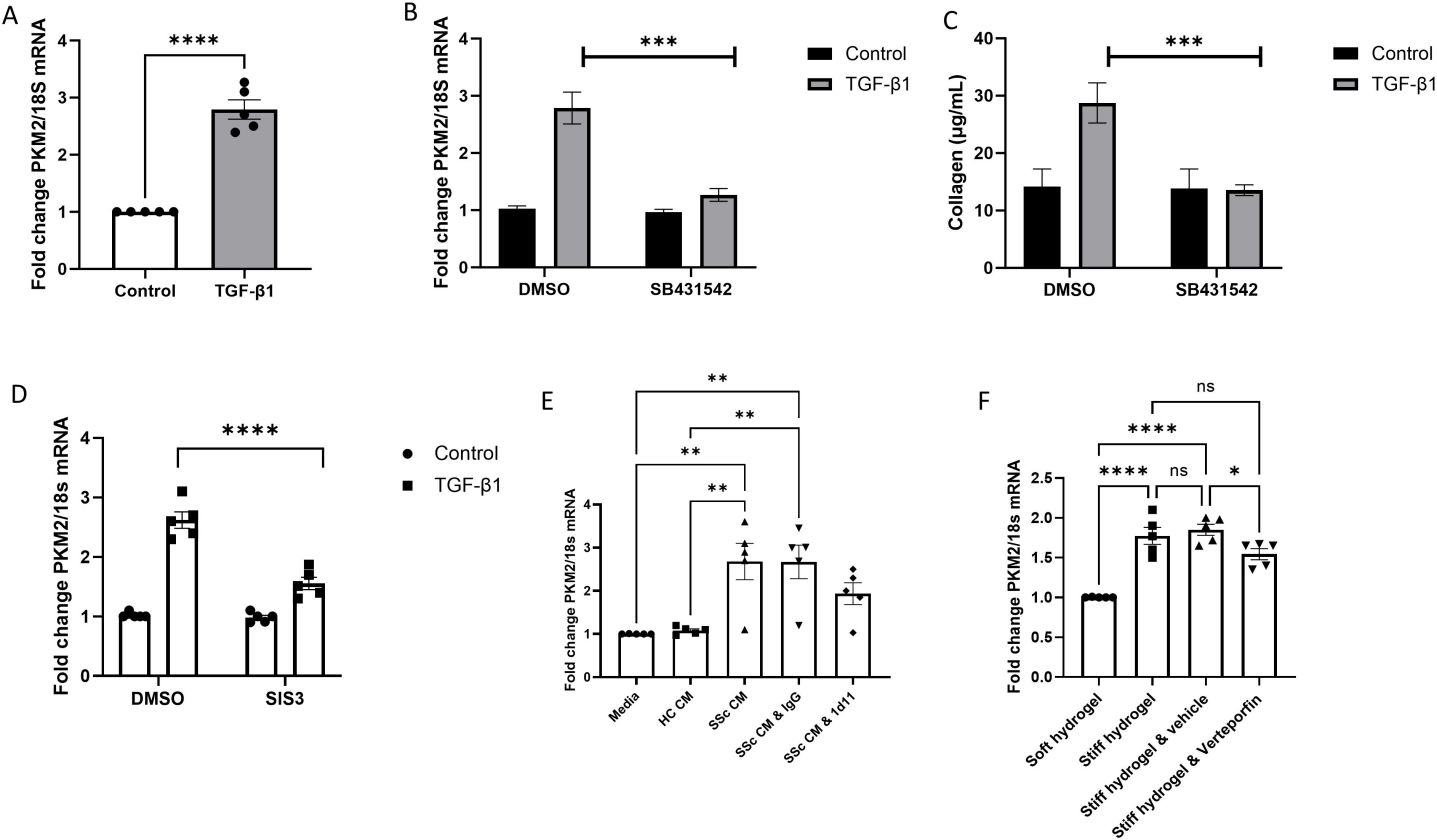
TGF-β1 induces PKM2 gene expression in dermal fibroblasts. **(A)** PKM2 was quantified by qPCR after 20 h of TGF-β1 (10 ng/ml) stimulation. Data are normalised to the 18S gene and presented as fold change relative to control (^****^p ≤ 0.0001). Data represent the mean and SEM (*n* = 5). **(B)** PKM2 expression was quantified by qPCR after DMSO vehicle control or SB431542 (10 μM) treatment, with or without TGF-β1 (10 ng/ml) for a further 20 h. Data are normalised to the 18S gene and shown as fold change relative to DMSO control cells. Data represent the mean and SEM (^***^p ≤ 0.001; *n* = 5). **(C)** Collagen release was quantified by Sircoll assay following incubation with DMSO or SB431542, with or without TGF-β1 (10 ng/ml). Data represent the mean and SEM (^***^p ≤ 0.0001, Student’s *t*-test; *n* = 5). **(D)** PKM2 gene expression was quantified by qPCR after DMSO (0.1%) vehicle or a specific inhibitor of Smad3, SIS3 (3 μM), with or without TGF-β1 (10 ng/ml). Data represent the mean and SEM (^****^p ≤ 0.0001, Student’s *t*-test; *n* = 5). **(E)** PKM2 gene expression was quantified by qPCR after incubation with media, HC CM, SSc CM, SSc CM with 1d11 (1 μg/ml), or SSc CM with IgG antibody (1 μg/ml) for 48 h. Data are normalised to the 18S gene and presented as fold change relative to standard media. Data represent the mean and SEM (HC vs. SSc CM: ^**^p = 0.0053; media vs. SSc CM: ^**^p = 0.0034; media vs. SSc CM IgG: ^**^p = 0.0036; one-way ANOVA multiple comparisons; *n* = 5). **(F)** PKM2 gene expression was quantified by qPCR after culture on soft or stiff hydrogels in the presence or absence of vehicle or veteporfin (10 μM). Data are normalised to the 18S housekeeping gene and presented as fold change relative to soft hydrogel alone cultures. Data represent the mean an SEM (soft vs. stiff hydrogel: ^****^p ≤ 0.0001; soft vs. stiff hydrogel plus vehicle: ^****^p ≤ 0.0001; soft hydrogel vs. stiff hydrogel plus verteporfin: ^***^p = 0.0004; stiff hydrogel plus vehicle vs. stiff hydrogel plus verteporfin: ^***^p = 0.0401; one-way ANOVA with multiple comparisons and Tukey’s correction, *n* = 5).

TGF-β1 is one of the most potent profibrotic molecules and is considered a prototypical fibrotic cytokine elevated in many fibrotic diseases, including SSc (1, 2, 23). To test the hypothesis that TGF-β1 regulates PKM2 levels, healthy dermal fibroblasts were incubated with TGF-β1 (10 ng/ml), which led to a significant upregulation of PKM2 mRNA (1.0 vs. 2.8-fold change; *p* ≤ 0.0001, Student’s *t*-test; *n* = 5) (**Figure 2A**). TGF-β1 normally signals through its receptors, activating intracellular cascades that involve Smad3 phosphorylation and nuclear translocation. To determine whether PKM2 induction was receptor-mediated, fibroblasts were pretreated with TGF-β1 receptor inhibitor SB431542 (10 µM). Pretreatment significantly attenuated TGF-β1-inguced PKM2 expression (*p* ≤ 0.0001, Student’s *t*-test; *n* = 5) (**Figure 2B**). This was also associated with a significant reduction in collagen release (**Figure 2C**). To further delineate the downstream signalling mechanism underlying TGF-β-mediated upregulation, we employed the specific Smad3 inhibitor SIS3, a small-molecule inhibitor of Smad3 (21). Dermal fibroblasts incubated with SIS3 (3 μM) exhibited significantly attenuated PKM2 induction by TGF-β1, indicating that Smad3 is important in mediating PKM2 expression in dermal fibroblasts (*p* = 0.0001, Student’s *t*-test; *n* = 5) (**Figure 2D**). We also cultured dermal fibroblasts from patients with SSc and collected conditioned media, which were subsequently applied to healthy dermal fibroblasts. After 48 h, PKM2 gene expression was evaluated in the recipient cells. Conditioned media from SSc fibroblasts induced a 2.6-fold increase in PKM2 expression compared with conditioned media from healthy controls (*p* = 0.0053, one-way ANOVA; *n* = 5) (**Figure 2E**). Incubation of SSc-conditioned media with the 1d11 antibody, which effectively neutralises TGF-β1 and β2, showed a trend towards reduced PKM2 induction, although this did not reach statistical significance. An isotype-matched antibody had no effect on PKM2 induction. To further investigate whether mechanical tension regulates PKM2, dermal fibroblasts were cultured on soft (0.6 kPa) and stiff (6 kPa) hydrogels that mimic physiological or pathological sclerotic situations. Cells grown on stiff hydrogels exhibited increased PKM2 gene expression, which was reduced by treatment with the YAP inhibitor verteporfin (**Figure 2F**). Statistical analysis confirmed significant differences (soft vs. stiff hydrogel: *p* < 0.0001; soft vs. stiff hydrogel plus verteporfin: *p* = 0.0004; stiff hydrogel plus vehicle vs. stiff hydrogel plus verteporfin: *p* = 0.0401; one-way ANOVA; *n* = 5). These findings suggest that mechanical tension governs PKM2 expression in dermal fibroblasts.

### Forced tetramerisation of PKM2 reduces fibrosis in SSc fibroblasts

Given that PKM2 expression is elevated in dermal fibroblasts from patients with SSc and is upregulated in healthy fibroblasts by TGF-β1, we hypothesised that PKM2 contributes to fibrosis and ECM deposition—hallmarks of SSc. To test this, we used TEPP-46, an allosteric regulator of PKM2 that promotes tetramer formation from the dimeric state by binding to a pocket at the PKM2 subunit interface (24).

**Figure 3:**
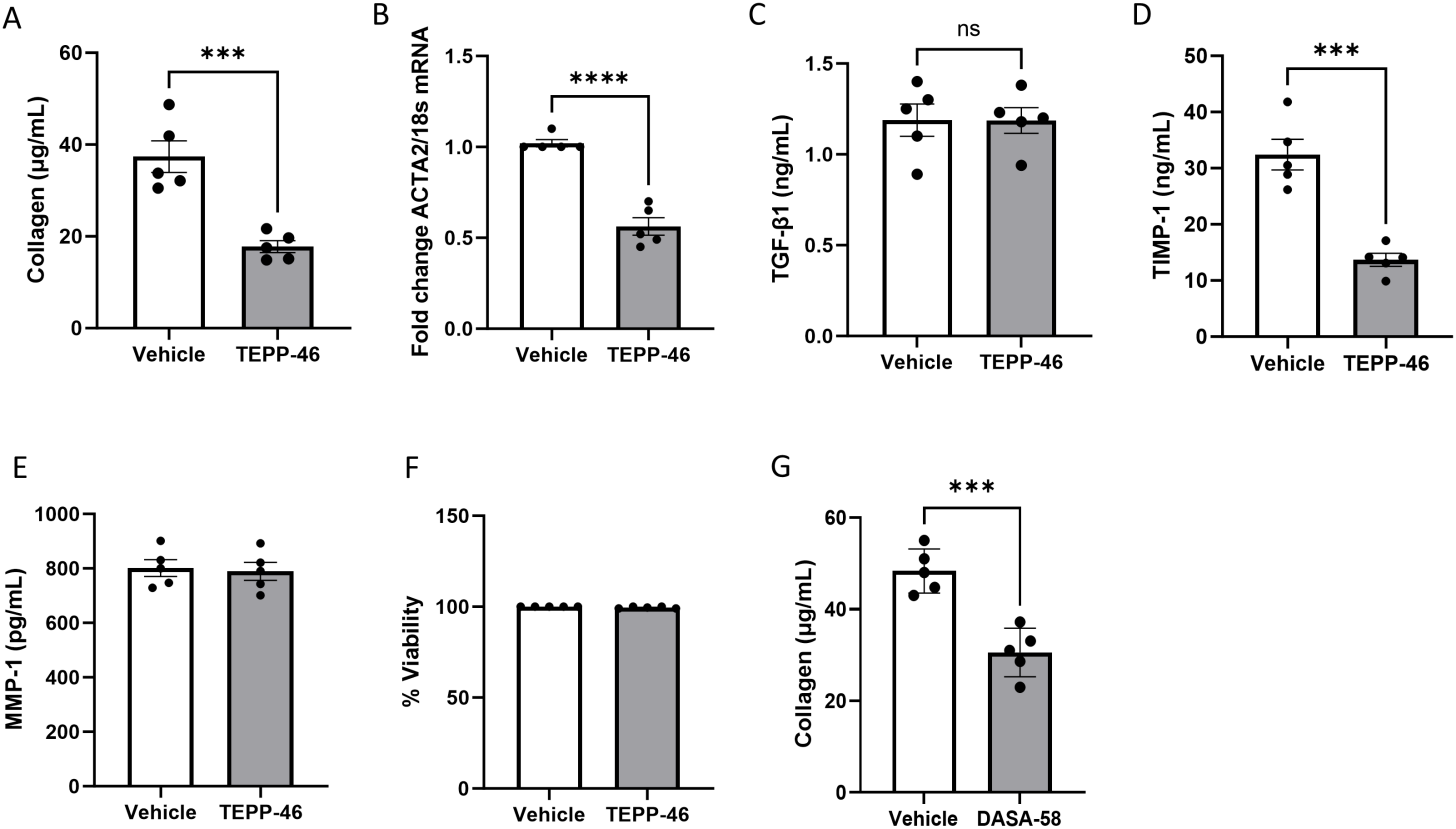
PKM2 modulation in SSc fibroblasts reduced ECM. **(A)** Collagen release in SSc dermal fibroblasts after TEPP-46 or vehicle was quantified following 30 h of incubation (^***^p = 0.0007, Student’s *t*-test; *n* = 5). Data are shown as individual points with mean and SEM. **(B)** ACTA2 was quantified by qPCR after vehicle or TEPP-46 treatment for 30 h (^****^p ≤ 0.0001, Student’s *t*-test; *n* = 5). **(C)** TGF-β1 was measured by ELISA after vehicle or TEPP-46 incubation (*p* = 0.98, Student’s *t*-test). Data are shown as individual points with mean and SEM (*n* = 5). **(D)** TIMP-1 was quantified by ELISA after vehicle or TEPP-46 treatment for 30 h (^***^p ≤ 0.0002, Student’s *t*-test). Data represent the mean and SEM (*n* = 5). **(E)** MMP-1 was quantified by ELISA after vehicle or TEPP-46 treatment for 30 h (*p* = 0.79, Student’s *t*-test). Data are shown as individual points with mean and SEM (*n* = 5). **(F)** Cell viability was measured after vehicle or TEPP-46 treatment and expressed as percentage viability relative to vehicle control set at 100% (*p* = 0.056). Data represent the mean and SEM (*n* = 5). **(G)** Collagen quantification in SSc fibroblasts was performed after vehicle or DASA-58 (10 μM) exposure for 30 h of stimulation (^***^p = 0.0005, Student’s *t*-test). Data represent the mean and SEM (*n* = 5).

PKM2 is primarily found in its dimeric form, which can translocate to the nucleus to regulate the expression of multiple target genes and exhibits lower kinase activity (13). Interestingly, JMJD5 has been shown to regulate nuclear translocation of PKM2 (25). Incubation of SSc dermal fibroblasts with TEPP-46 significantly reduced release of collagen release with vehicle-treated cells (37.4 vs. 17.8 μg/ml; *p* = 0.0007, Student’s *t*-test; *n* = 5) (**Figure 3A**). Moreover, the classic myofibroblast marker ACTA2, encoding α-smooth muscle actin, was significantly reduced in TEPP-46-treated cells compared with vehicle controls (1.0 vs. 0.56-fold change; *p* ≤ 0.0001, Student’s *t*-test; *n* = 5) (**Figure 3B**). No difference in TGF-β1 expression was observed in TEPP-46-treated fibroblasts compared with vehicle controls (*p* = 0.98, Student’s *t*-test) (**Figure 3C**), suggesting that PKM2 does not directly regulate TGF-β1 expression. TIMP-1, the major inhibitor of ECM degradation via the inhibition of matrix proteases, was significantly reduced in TEPP-46-treated cells (32.4.8 vs. 13.7 ng/ml; *p* ≤ 0.0002, Student’s *t*-test; *n* = 5) (**Figure 3D**).

As TIMP-1 is the primary inhibitor of MMP-1, we next assessed whether MMP-1 levels were altered. TEPP-46 treatment did not affect MMP-1 expression (*p* = 0.79, Student’s *t*-test; *n* = 5) (**Figure 3E**), indicating that the TIMP-1/MMP-1 expression ratio remained unchanged. Cellular viability was also assessed to determine whether PKM2 modulation exhibited cytotoxic effects on fibroblasts; however, no significant difference in viability was observed (*p* = 0.056, Student’s *t*-test; *n* = 5) (**Figure 3F**). To further confirm the role of PKM2 in SSc, fibroblasts were incubated with DASA-58, another highly selective PKM2 activator (24). DASA-58 treatment significantly reduced collagen production in SSc fibroblasts (*p* = 0.0005, Student’s *t*-test) (**Figure 3G**).

### PKM2 governs glycolysis, and PKM2-mediated lactate regulates ECM

**Figure 4:**
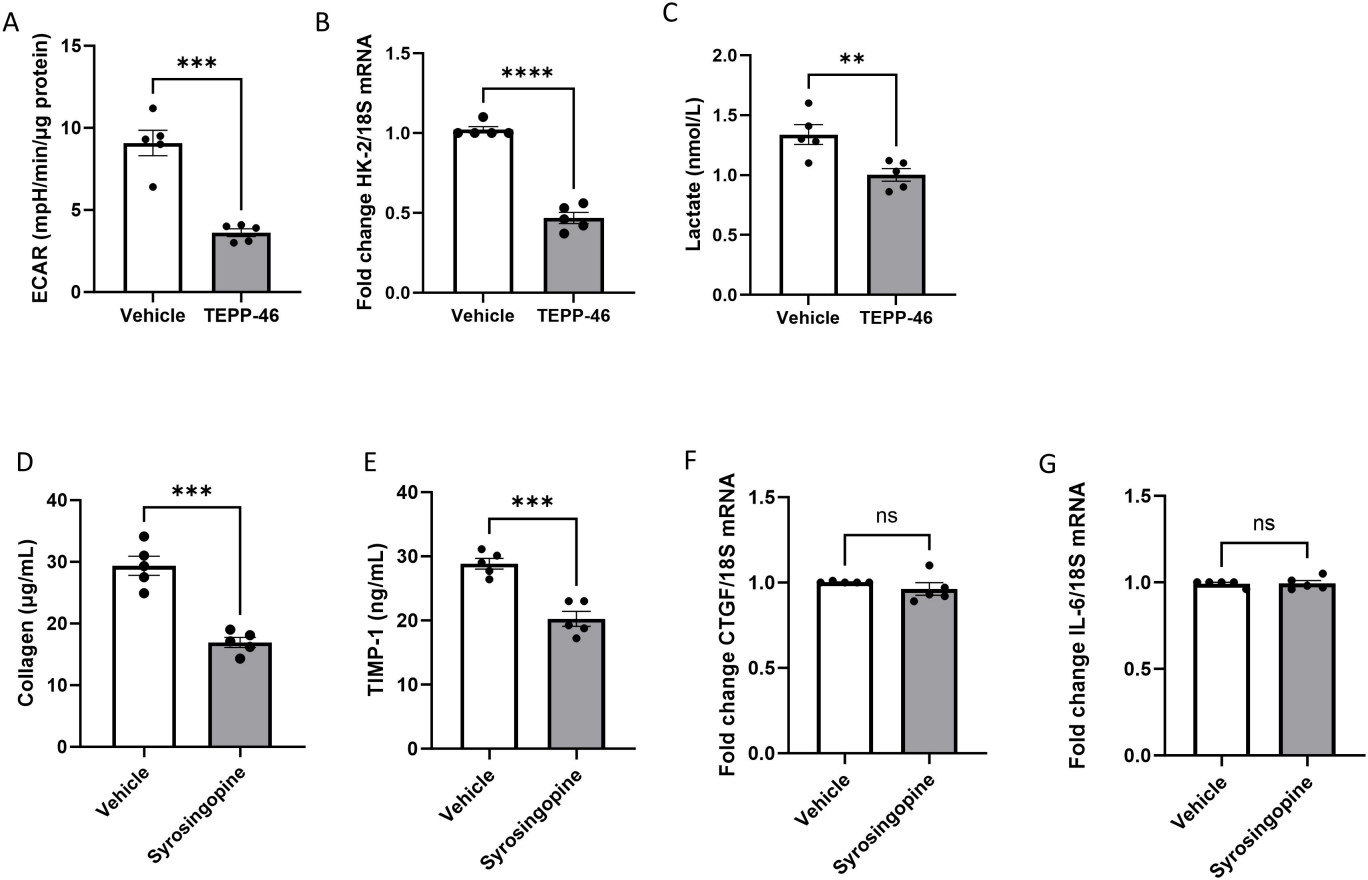
PKM2 tetramisation reduced glycolysis. **(A)** ECAR was quantified using a Seahorse analyser after stimulation with TGF-β1 (10 ng/ml) in all cultures and either vehicle or TEPP-46 (40 μM) (^***^p = 0.001, Student’s *t*-test). Data represent the mean and SEM (*n* = 5). **(B)** HK2 gene expression was analysed by qPCR. Data are normalised to 18S gene expression and presented as fold change relative to vehicle control (^****^p ≤ 0.0001, Student’s *t*-test; *n* = 5). **(C)** Extracellular lactate was quantified in the media using a commercial lactate kit (^**^p = 0.0019, Student’s *t*-test). Data represent the mean and SEM (*n* = 5). **(D)** Collagen secretion was quantified after vehicle or syrosingopine (10 μM) exposure in the presence of TGF-β1 after 48 h (^***^p = 0.0001, Student’s *t*-test; *n* = 5). **(E)** TIMP-1 was quantified by ELISA after vehicle or syrosingopine (10 μM) exposure in the presence of TGF-β1 after 48 h (^***^p = 0.0003, Student’s *t*-test; *n* = 5). **(F)** CTGF expression was analysed after vehicle or syrosingopine (10 μM) exposure in the presence of TGF-β1 after 48 h. Data are normalised to 18S gene expression and presented as fold change relative to vehicle control (*p* = 0.309, Student’s *t*-test; *n* = 5). **(G)** IL-6 gene expression was analysed after vehicle or syrosingopine (10 μM) exposure in the presence of TGF-β1 after 48 h (*p* = 0.9150, Student’s *t*-test; *n* = 5); ns, nonsignificant.

How does PKM2 in its dimeric form lead to ECM deposition? In its dimeric form, PKM2 can drive the so-called Warburg effect (24, 26), resulting in increased aerobic glycolysis. In its tetrameric form, PKM2 reduces aerobic glycolysis (27). Given the recent reports on the role of glycolysis in mediating fibrosis (5, 28) and, in particular, SSc (6, 29), we proposed that the antifibrotic effects of PKM2 tetramerisation are mediated by reduced glycolysis and its metabolites. Therefore, we measured the ECAR as a readout of glycolysis after TEPP-46 or vehicle treatment in fibroblasts exposed to TGF-β1. **Figure 4A** demonstrates that the ECAR was significantly reduced in TGF-β1-exposed fibroblasts with TEPP-46 incubation (9.1 vs. 3.6 mpH/min/μg; *p* = 0.0001, Student’s *t*-test; *n* = 5). HK-2 is a critical rate-limiting enzyme in glycolysis, and we have previously demonstrated its upregulation in SSc (6); therefore, we measured its expression in TEPP-46-treated cells. It was found that TEPP-46 reduced HK-2 gene expression by over 52% compared to vehicle-treated cells (*p* ≤ 0.0001, Student’s *t*-test; *n* = 5) (**Figure 4B**). One of the main products of glycolysis is lactate. Lactate is generated by the enzyme lactate dehydrogenase-A (LDH-A), and lactate can modify histone proteins posttranslationally (28, 30). We therefore quantified lactate levels in these fibroblasts. **Figure 4C** demonstrates that cells treated with TEPP-46 had significantly lower lactate levels compared with vehicle-treated cells (**Figure 4C**).

Given that lactate is a significant byproduct of glycolysis, and PKM2 lies upstream of this pathway and was reduced by TEPP-46, we hypothesised that PKM2-mediated lactate may drive ECM deposition. The main cellular transporter for lactate is the lactate importer monocarboxylate transporter-1 (MCT-1), which transports lactate across the plasma membrane. To evaluate whether lactate is important in this process, we used the MCT-1-specific inhibitor syrosingopine (10 μM). Fibroblasts treated with TGF-β1 plus syrosingopine had reduced collagen expression compared with vehicle-treated cells (*p* = 0.0001, Student’s *t*-test; *n* = 5) (**Figure 4D**). This was also associated with reduced TIMP-1 expression following MCT-1 inhibition *(p* = 0.0003, Student’s *t*-test; *n* = 5) (**Figure 4E**). There was also a small but nonsignificant reduction in CTGF expression levels (*p* = 0.309, Student’s *t*-test; *n* = 5) (**Figure 4F**), and at the mRNA level, there was no change. As lactate has also been found to alter immune function, we measured IL-6 levels after lactate transport inhibition, but no significant difference was observed (*p* = 0.9150, Student’s *t*-test; *n* = 5) (**Figure 4G**).

### PKM2-facilitated lactate promotes collagen in fibroblasts through lactylation and HIF-1α modulation

**Figure 5:**
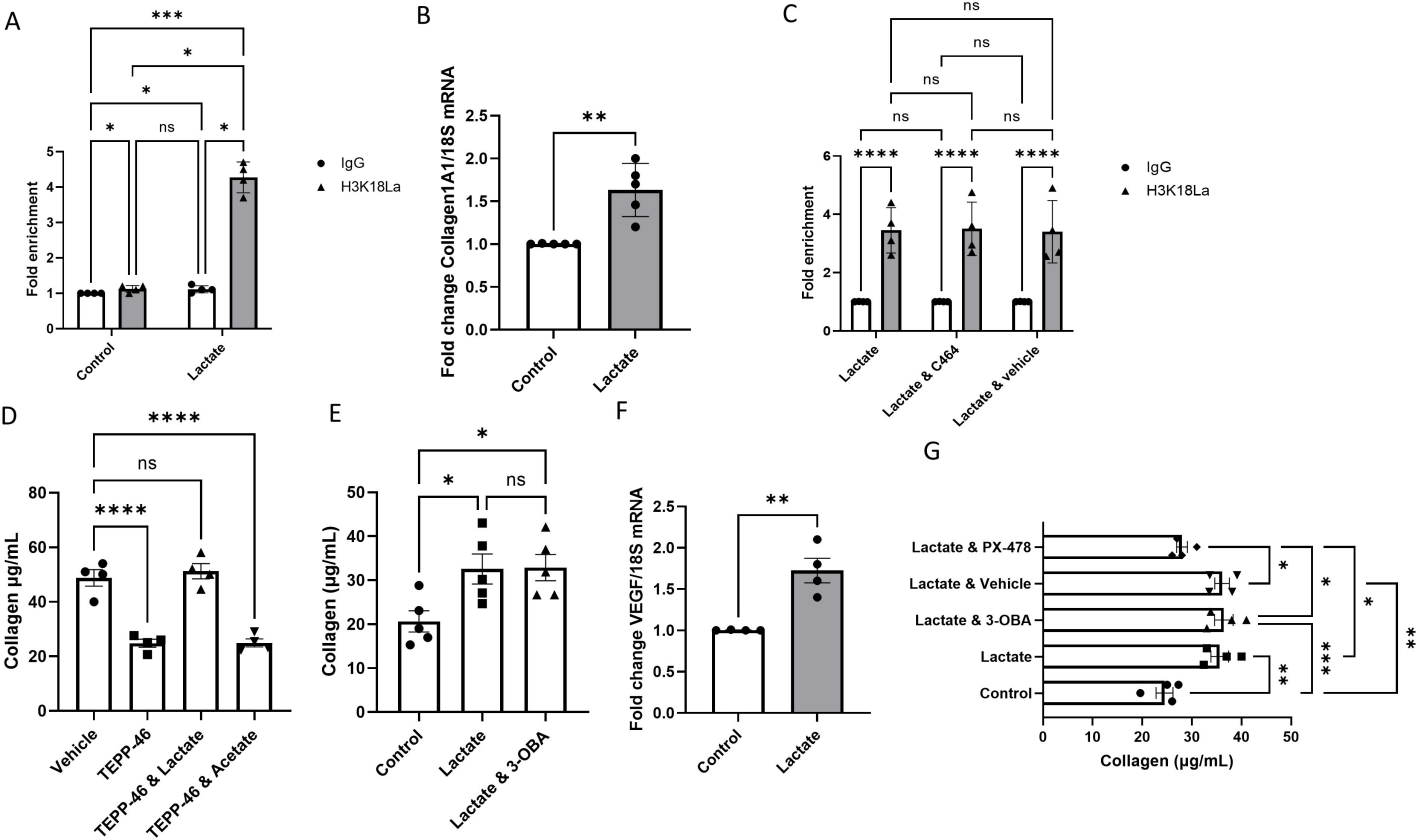
Lactate mediates collagen expression. **(A)** Fold enrichment at the collagen1A1 promoter compared with IgG control antibody precipitation was quantified after lactate (20 mM) treatment for 20 h in primary healthy dermal fibroblasts. Control IgG vs. lactate H3K18La (^***^p = 0.0007); control H3K18La vs. lactate H3K18La (^*^p = 0.0113); lactate IgG vs. lactate H3K18La (^*^p = 0.0108); ns, nonsignificant. Two-way ANOVA with Tukey’s correction. Data represent the mean and SEM (*n* = 4). **(B)** Collagen gene expression was quantified in dermal fibroblasts after lactate (20 mM) treatment for 24 h. Data are normalised to 18S gene expression and presented as fold change relative to untreated cultures (^**^p = 0.0019, Student’s *t*-test, *n* = 5). **(C)** Fold enrichment at the collagen1A1 promoter compared with IgG control antibody precipitation was quantified after C646 (1.2 μM) treatment or DMSO vehicle exposure for 1 h, followed by Na-l-lactate (20 mM) for a further 20 h in primary healthy dermal fibroblasts. Lactate IgG vs. H3K18La (^****^p ≤ 0.0001); lactate and C464 IgG vs. lactate and C464 H3K18La (^****^p = 0.0001); lactate and vehicle IgG vs. lactate and vehicle H3K18La (^****^p ≤ 0.0001); ns, nonsignificant. Two-way ANOVA with Tukey’s correction. Data represent the mean and SEM (*n* = 4). **(D)** Collagen protein was quantified in SSc dermal fibroblasts treated with vehicle, TEPP-46, TEPP-46 plus lactate (20 mM), or acetate (20 mM) (*n* = 4 SSc donors; ns). Vehicle vs. TEPP-46 (^****^p ≤ 0.0001); vehicle vs. TEPP-46 plus acetate (ns, nonsignificant). One-way ANOVA with Bonferroni correction (*n* = 4). **(E)** HC dermal fibroblasts were treated with lactate (20 mM) or the GPR81 antagonist 3-OBA (500 nM), and collagen was quantified. Control vs. lactate (^*^p = 0.037); control vs. lactate and 3-OBA (^*^p = 0.035); ns, nonsignificant. One-way ANOVA with Tukey’s correction (*n* = 5). **(F)** VEGF was quantified by qPCR after lactate (20 mM) treatment for 24 h. Data are normalised to 18S gene expression and presented as fold change relative to untreated fibroblasts (^**^p = 0.0029, Student’s *t*-test; *n* = 4). **(G)** Dermal fibroblasts were quantified for collagen after treatment with lactate (20 mM), lactate plus 3-OBA (GPR81 inhibitor), or PX-478 (HIF-1α inhibitor), or vehicle. Control vs. lactate (^**^p = 0.0019); control vs. lactate plus 3-OBA (^***^p = 0.0009); control vs. lactate plus vehicle (^**^p = 0.0013); lactate vs. lactate plus PX-478 (^*^p = 0.0441); ns, none significant. One-way ANOVA with Bonferroni’s correction (*n* = 4).

Lactate is now recognised as a histone-associated epigenetic posttranslational modification capable of mediating multiple downstream effects, with established roles in cancer development and wound healing (30). This epigenetic modification leads to permissive gene expression. Using chromatin immunoprecipitation with a specific antibody against the lysine lactylation mark H3K18La and primers targeting the collagen1A1 promoter, we observed a significant enrichment over the IgG control antibody (**Figure 5A**) following Na-lactate treatment (*p* = 0.001, two-way ANOVA).

This was coincident with elevated gene expression of collagen in response to lactate exposure (*p* = 0.0019, Student’s *t*-test) (**Figure 5B**). Since lactic acid may change the pH to a more acidic environment, we adjusted DMEM (the primary medium) to pH 6.5 to determine whether the increase was due to acidity *per se*, and saw no increase in collagen (data not shown). It has previously been reported that the acetyltransferase P300/CBP is responsible for facilitating the transfer of lactate onto histone lysine residues, in a similar fashion to its transfer of acetyl groups (30). We therefore questioned the role of P300/CBP in mediating the enrichment at the collagen1A1 promoter. Pretreatment with the specific P300/CBP inhibitor C646 did not reduce the enrichment at the promoter following lactate treatment in dermal fibroblasts, suggesting that P300/CBP is unlikely to be the responsible transferase (**Figure 5C**).

Having established the key role of PKM2 in mediating fibrosis in dermal fibroblasts and recognising that lactate is the terminal product of glycolysis, we used dermal fibroblasts from SSc donors and incubated them with TEPP-46 to tetramerise PKM2, resulting in reduced collagen that, importantly, could be restored with the addition of exogenous lactate, but notably, not acetate (**Figure 4D**). Acetate was chosen as it can also modify histones, leading to changes in protein expression (31). Lactate has been suggested to bind to a lactate-specific receptor called GPR81; upon binding, it elicits downstream signalling through Cyclic Adenosine Monophosphate (cAMP) modulation (32), the effect of which is cell-type dependent. We therefore tested the effect of inhibiting GPR81 by using a specific antagonist. We found that inhibiting GPR81 with 3-OBA did not diminish lactate-induced collagen production (lactate vs. lactate plus 3-OBA; one-way ANOVA) (**Figure 5E**). This suggests that the effect of lactate is not facilitated through the GPR81 receptor.

It was previously demonstrated in other systems that glycolysis-driven lactate can stabilise HIF-1α, leading to HIF-1α-dependent downstream effects (33). We hypothesised that lactate leads to HIF-1α stabilisation and ECM modulation. We exposed healthy dermal fibroblasts to lactate and measured the expression of a *bona fide* HIF-1α target gene, VEGF, which was significantly (1.73-fold) upregulated compared with untreated cells (*p* = 0.0029, Student’s *t*-test) (**Figure 5F**). Pretreatment with the general antioxidant *N*-acetyl cysteine (NAC) significantly suppressed the induction of the HIF-1α target gene VEGF (*p* = 0.0180, one-way ANOVA) (**Supplementary Figure 1**).

It has been suggested that ROS mediates stabilisation of HIF-1α in other cell types, and we observed increased expression of the HIF-1α target gene VEGF, which could be attenuated by NAC. We then determined the role of HIF-1α after lactate treatment. Using a small-molecule inhibitor of HIF-1α, we observed that inhibition of HIF-1α significantly inhibited the lactate-evoked upregulation of collagen in dermal fibroblasts (lactate vs. lactate plus PX-478, *p* = 0.44; control vs. lactate, *p* = 0.0019; one-way ANOVA; *n* = 4), whereas collagen expression was not affected by vehicle control or the GPR81 antagonist 3-OBA (**Figure 5G**), suggesting that HIF-1α is important in mediating lactate-evoked collagen induction.

### Lactate-mediated collagen is STAT3 independent

**Figure 6:**
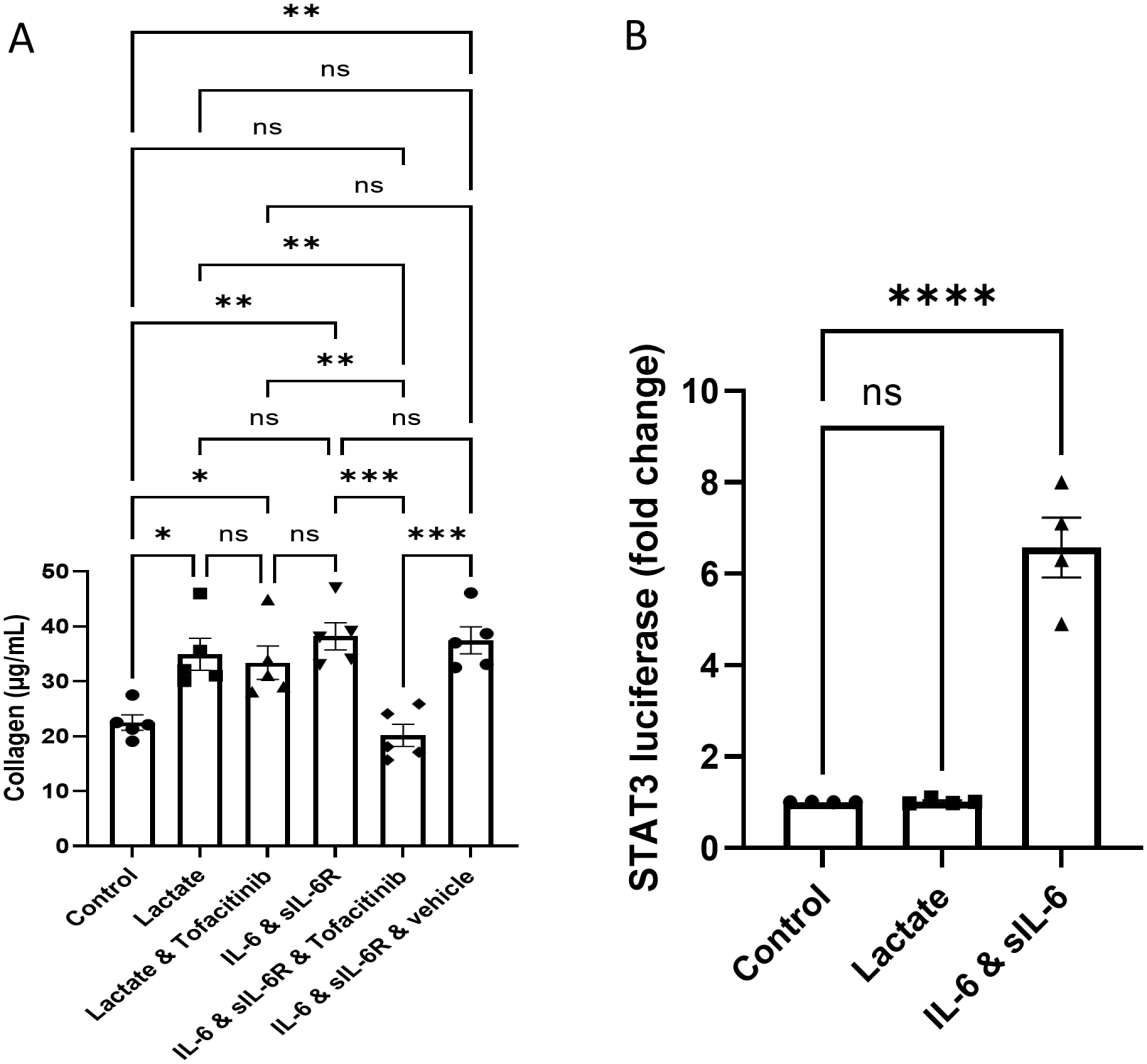
Lactate-induced collagen is independent of STAT3. **(A)** Healthy dermal fibroblasts were untreated or treated with lactate (20 mM), lactate plus tofacitinib (100 nM), IL-6 and sIL-6R, IL-6 and sIL-6R plus tofacitinib, or IL-6 and sIL-6R plus vehicle for 24 h, after which collagen was quantified. Control versus lactate (^*^p = 0.0162); control vs. lactate plus tofacitinib (^*^p = 0.0442); control vs. IL-6 and sIL-6R (^***^p = 0.0017); IL-6 and sIL-6R vs. IL-6 and sIL-6R plus tofacitinib (^***^p = 0.0003); IL-6 and sIL-6R plus tofacitinib vs. IL-6 and sIL-6R plus vehicle (^***^p = 0.0005); ns, nonsignificant. One-way ANOVA with Bonferroni’s correction (*n* = 5). Data represent the mean and SEM. **(B)** Luciferase activity of a STAT3 reporter was quantified after transfection into dermal fibroblasts. Cells were left untreated or treated with lactate or IL-6 and IL-6R for 12 h. Transfection efficacy was normalised to Renilla. Data are presented as fold change relative to untreated cells (^****^p ≤ 0.0001; ns, nonsignificant; one-way ANOVA; *n* = 4). Data represent the mean and SEM.

As lactate has also been suggested to activate STAT3 (34), a key transcription factor in various immune pathways and collagen regulation (35), we determined the role of STAT3 in lactate-evoked collagen production. Lactate-induced collagen was significantly increased compared to untreated cultures but was unaffected by tofacitinib treatment; however, IL-6 trans-signalling-induced collagen was significantly inhibited by tofacitinib (**Figure 6A**). This was also confirmed using a STAT3 luciferase reporter, where no signal was seen with lactate, but luciferase activity was increased with IL-6 trans-signalling (^****^p ≤ 0.0001; one-way ANOVA) (**Figure 6B**). Therefore, STAT3 is not involved in lactate-mediated collagen induction.

### PKM-2 in patients with SSc is modulated by immunomodulatory therapy

**Figure 7:**
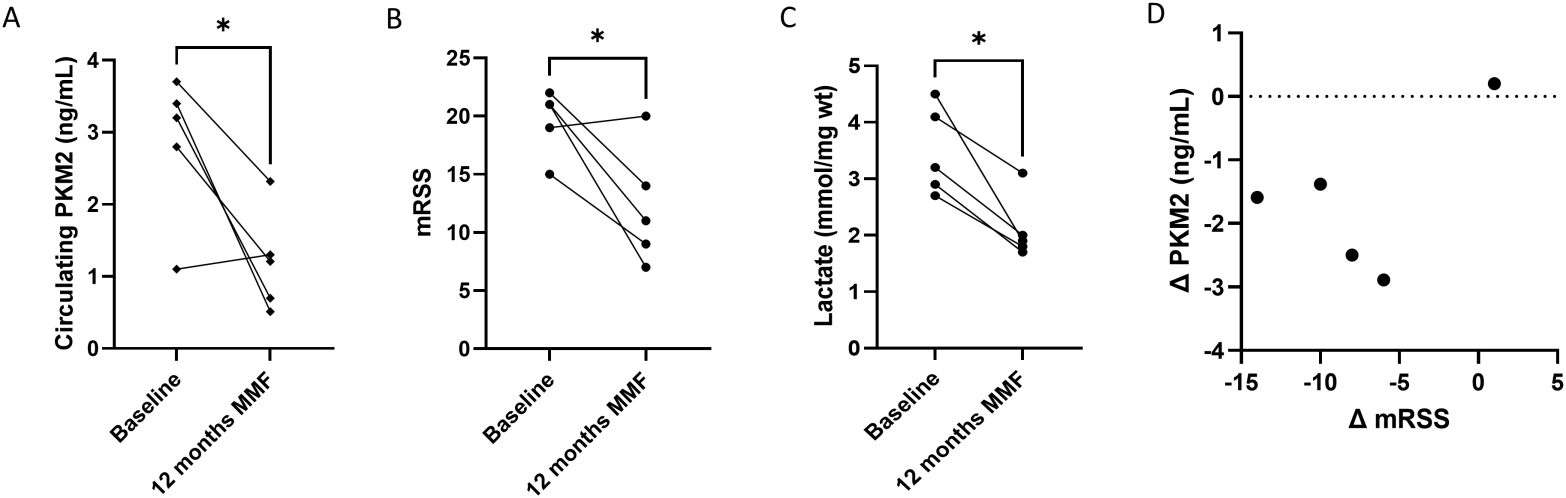
Reduction of PKM2 levels 12 months after MMF treatment with mRSS change. **(A)** Circulating PKM2 levels were measured at baseline and after 12 months of MMF treatment in five SSc patients. Data are shown as matched individual data points for each patient (^*^p = 0.0383, paired Student’s *t*-test; *n* = 5). **(B)** mRSS was evaluated at baseline and after 12 months of MMF treatment in five SSc patients (^*^p = 0.0407, paired Student’s *t*-test; *n* = 5). Note that the patient who did not respond was also the patient who did not exhibit a reduction in serum PKM2 levels. **(C)** Lactate was evaluated at baseline and after 12 months of MMR treatment in five SSc patients (^*^p = 0.0113, paired Student’s *t*-test; *n* = 5). **(D)** Correlation analysis between ΔPKM2 and ΔmRSS (*R*^2^ = 0.25; *p* = 0.40; Pearson’s correlation; *n* = 5). The dotted line indicates 0 on the *x*-axis.

Finally, we measured the levels of circulating PKM2 in sera from five patients with SSc at baseline and after 12 months of MMF treatment (< 2 g/day) and found that PKM2 levels were reduced in 80% (four of five) of the patients treated (*p* = 0.0383, Student’s *t*-test) (**Figure 7A**). This was accompanied by a clinical reduction in the mRSS scores of individual patients (mRSS range, 0–51), with a mean ΔmRSS of − 7/4 (SD: 5.5), and four of five patients having a significant reduction in mRSS (*p* = 0.0407, Student’s *t*-test; *n* = 5) (**Figure 7B**). Moreover, this reduction was clinically meaningful, being defined as over a 25% decrease in mRSS compared with the baseline skin score. This was accompanied by reductions in lactate levels in whole-skin biopsies from treated patients (**Figure 7C**). Correlation analysis failed to find a significant correlation between ΔPKM2 levels and ΔmRSS (*R*^2^ = 0.25, *p* = 0.40; *n* = 5) (**Figure 7D**). Similarly, no significant correlation was observed between ΔPKM2 levels and Δtissue lactate (*R*^2^ = 0.0004, *p* = 0.973, Pearson’s correlation; *n* = 5).

## Discussion

SSc is an autoimmune connective tissue disorder characterised by the activation of quiescent fibroblasts into myofibroblasts that secrete copious amounts of ECM, leading to fibrosis. We examined the expression of PKM2 in this disease and found that it is elevated in systemic sclerosis and is important in fibrosis development.

Recently, evidence has been accumulating, suggesting a role for metabolic reprogramming in fibrosis and, in particular, glycolysis in SSc development (6). To investigate this further, we examined the expression of PKM2, the rate-limiting enzyme in glycolysis that leads to lactate production and is a hallmark of cancer cells (36). We found significantly elevated levels of circulating serum PKM2 and elevated cellular levels of PKM2 in SSc. We also demonstrated that PKM2 can be induced by the classic profibrotic molecule TGF-β1 (23). This effect is mediated through classical TGF-β signalling mechanisms. Extracellular PKM2 has been found in a variety of diseases, including cancer (37). TGF-β1 is significantly associated with fibrotic disease, and it is elevated in SSc and plays a key role (23, 38). Therefore, we propose that TGF-β lies upstream of PKM2-mediated fibrosis. We determined that TGF-β1-mediated upregulation of PKM2 occurred through TGF-β receptor-dependent mechanisms and is, at least partially, dependent on SMAD3. SMAD3 is a key downstream effector of TGF-β signalling and translocates to the nucleus, where it directs gene expression programs and is heavily implicated in fibrosis. These data are in accordance with recent data demonstrating that in normal lung fibroblasts and the hepatic cell line LX-2, TGF-β1 stimulation significantly upregulates PKM2 expression concurrently with ECM deposition (17), although the authors did not determine the downstream signalling responsible for the effects of TGF-β1. Furthermore, we demonstrated that factors within the SSc fibroblasts’ secretome and mechanical tension drive increased PKM2 expression. On stiff hydrogels, fibroblasts increased the expression of PKM2, which could be blocked with the YAP inhibitor verteporfin. YAP, in association with TAZ, is a key mechanosensitive protein that can mediate mechanical signals into transcriptional activation, and YAP inhibition is antifibrotic in SSc fibroblasts and in the bleomycin mouse model of fibrosis (39, 40). Indeed, we suggest that stiff surfaces resembling pathological sclerotic tissue promote PKM2 and glycolysis, forming a feed-forward loop in fibrosis. In breast cancer cells, it has been demonstrated that YAP drives glycolysis and, in this case, tumorigenesis (41). Furthermore, YAP promotes glycolysis by directly inducing the key glycolytic enzyme hexokinase II, thereby enhancing glycolysis (42).

PKM2 is a critical enzyme that catalyses the final step of the glycolytic pathway by catalysing the transfer of a phosphate group from PEP to adenosine diphosphate (ADP), producing pyruvate, and exists in two states: the dimeric or tetrameric form (26).

In its dimeric form, PKM2 has lower pyruvate kinase activity but can promote aerobic glycolysis and translocate to the nucleus to regulate gene expression (11); furthermore, other so-called moonlighting functions of dimeric PKM2 have been described, including acting as a protein kinase (43). In particular, in its dimeric form, PKM2 has been found to be preferentially used in highly proliferating cells, such as cancer cells, where it creates biosynthetic intermediates derived from glycolysis to support cellular growth required for tumour development (44).

It has been shown by us and others that aerobic glycolysis facilitates the transition of fibroblasts to myofibroblasts (45), and we have used broad glycolytic inhibitors and demonstrated reduced ECM production (6). Aside from its role in glycolysis and the so-called Warburg effect, PKM2 has functions in regulating gene expression and can increase the expression of specific target genes via phosphorylating the transcription factor STAT3, only when in its dimeric form (43). We proposed that PKM2 mediates fibrosis in its dimeric form, as opposed to its tetrameric form. To this end, we used the small molecule TEPP-46, which promotes the tetramerisation of PKM2 via enhanced association of PKM2 subunits. Using TEPP-46 in SSc fibroblasts demonstrated that PKM2 tetramerisation reduced collagen secretion, alpha-smooth muscle actin expression, and TIMP-1 expression. However, MMP-1 levels, the matrix-degrading enzyme, were not affected; thus, the balance between matrix deposition and degradation is skewed towards reduced matrix deposition.

It was demonstrated in LX-2 hepatic cells that PKM2 dimerisation reduced markers of myofibroblast differentiation, such as collagen and alpha SMA, and this was also recapitulated in human lung fibroblasts (17). From a mechanistic point of view, it appeared that PKM2 dimerisation drove myofibroblast differentiation by increasing serine–glycine metabolism, thereby providing the substrates for collagen synthesis (17). Furthermore, in an animal model of bleomycin-induced lung fibrosis, PKM2 tetramerisation with *in vivo* TEPP-46 treatment led to reduced lung scarring and fibrosis (17). Furthermore, TEPP-46-induced PKM2 tetramer formation blocks the activation of mouse and human hepatic myofibroblasts *in vitro* (46). This was also protective against liver fibrosis in preclinical models of liver fibrosis, including carbon tetrachloride models (46). Mechanistically, it was demonstrated that PKM2 dimers bound the promoters of MYC and CCND1, via histone acetylation changes, to drive fibrosis, and TEPP-46 or genetic depletion of PKM2 reduced the induction of these genes *in vivo* (46). Both MYC and CCND1 are important drivers of hepatic fibrosis (47). Furthermore, TEPP-46 reduced cardiac fibrosis in a mouse model of cardiac fibrosis by tetramerisation of PKM2 (48). We used a different PKM2 activator, DASA-58, which has been reported to be a PKM2-specific tetramerising agent (24), and observed reduced collagen expression in SSc cells upon exposure, demonstrating that PKM2 allosteric regulation is important in fibrosis. DASA-58 has also been found to inhibit stellate cell activation and thereby attenuate liver fibrosis. Interestingly, shikonin has been reported to inhibit PKM2 activity but may not mediate this in a specific way; in other words, it may not preferentially activate tetramer formation over dimer formation and may block PKM2 nonspecifically. *In vitro*, hypertrophic scar fibroblasts, similar to SSc fibroblasts, exposed to shikonin showed reduced collagen expression (49).

Given that the tetramerisation of PKM2 appears to be antifibrotic, we sought to understand the underlying mechanism. Lactate is the main product of glycolysis, and glycolysis has been associated with metabolic reprogramming in multiple contexts, including fibrosis (6) and pulmonary fibrosis (45). We predicted that lactate may play a role in mediating the transition of fibroblasts to myofibroblasts. MCT-1 is the main lactate transporter in the cell membrane (50); blocking this led to significant reductions in ECM. Stellate cell targeting of MCT-1 reduced liver fibrosis in a mouse model of fibrosis, and siRNA targeting of MCT-1 in human LX-2 cells reduced collagen expression (51). It has been shown that lactate has a variety of functions, including modifications of the tumour microenvironment and signalling in immune cells (52). It has been shown to modulate T-cell function and to activate latent TGF-β into its active form. Kottmann et al. demonstrated that lactate activated TGF-β1 in a pH-dependent manner to promote myofibroblast activation; blockade of lactate led to reduced ECM deposition and myofibroblast differentiation (53). They further demonstrated that, in the bleomycin model of lung fibrosis, pharmacological blockade of lactate production led to reduced lung fibrosis associated with less active TGF-β1 (54). Lactate has been found to be elevated in IPF lungs, with dysregulation of lactate dehydrogenase (55). Furthermore, we have found higher levels of lactate in the blister fluid of SSc patients compared to healthy controls (56), suggesting that lactate can promote myofibroblast transition; we have also observed elevated LDH expression (6). The novel posttranslational modification called lactylation regulates gene expression epigenetically and is associated with wound healing and oncogenesis (30). Using ChIP, we demonstrated lactylation at the promoter region of the collagen1A1 gene, strongly suggesting that lactylation is important in fibrosis development. It has been shown that, in lung fibrosis, myofibroblasts release lactate that drives profibrotic responses in lung macrophages by histone lactylation and that this is mediated by the enzyme P300 (28). P300 has been claimed to be the enzyme that mediates the addition of the lactate group to histones, and to test this hypothesis, we treated cells with a P300 inhibitor, but we did not find a reduction in enrichment at the collagen promoter, strongly suggesting that this is not the enzyme responsible for lactylation. However, recently, other proteins have been identified that mediate lactylation, including alanyl-tRNA synthetase (AARS1) and HDAC6 (57, 58), and it could be these, or other as yet unidentified, transferases that mediate the effect. Since we identified lactate as a key molecule and PKM2 lies upstream of lactate in the glycolytic pathway, we used SSc dermal fibroblasts and inhibited PKM2 and demonstrated reduced enrichment at the collagen promoter, which, importantly, could be restored with exogenous lactate but not acetate. Acetate can also be added to histones to mediate gene expression, suggesting that this is not facilitating ECM. Exogenous lactate also did not lead to elevated TGF-β1 levels, suggesting that this is not playing a role.

It has been suggested that lactate has a specific receptor through which it mediates its effect, called GPR81, and can elicit cAMP signalling (32). We used the GPR81 antagonist 3-OBA to determine the role of this receptor in the effects of lactate, and we demonstrated that this receptor is not involved in mediating the collagen effects of lactate. It has been shown that lactate can stabilise the transcription factor HIF-1α to elicit its effects (33), so we determined that the HIF-1α target gene *VEGF* was elevated by lactate and could be blocked by NAC, implying that it is mediated via ROS, in keeping with other studies (33). HIF-1α stabilisation in the absence of hypoxia has been termed “pseudohypoxia”. Mechanistically, increased ROS inhibits prolyl hydroxylase domain protein 2 (PHD2) (59). PHD2 normally hydroxylates HIF-1α, marking it for degradation; thus, its reduction would lead to enhanced stability of HIF-1α. Importantly, lactate-induced collagen expression was inhibited by the specific HIF-1α inhibitor, but not GPR81 inhibition, demonstrating that lactate mediates collagen induction via HIF-1α, at least partially. Distler et al. had previously shown elevated HIF-1α in SSc whole skin and fibroblasts (19). We also excluded a role for STAT3 in lactate-mediated collagen induction, as lactate had been demonstrated to activate a STAT3 programme in T cells (34), and tofacitinib did not block the induction of collagen; as a control, tofacitinib did retard trans-signalling-mediated collagen, as previously described (35). We used a STAT3 luciferase reporter to confirm this.

We also demonstrated that, in five patients from the cohort who were treated with the immunomodulator MMF, there were significant reductions in serum PKM2, mRSS, and lactate in whole tissue. mRSS is a measure of skin thickness, and four of the five donors showed a reduction of more than 25%, which is deemed clinically meaningful. It is interesting that tissue lactate concentration also declined in these patients. We have previously demonstrated elevated lactate in the blister fluid of SSc patients (56). This suggests that PKM2 could be a marker of response to therapy and could be modulated. However, when examining correlations between the ΔPKM2 levels and ΔmRSS, no significant correlation was apparent, but this could be due to the small sample size in this treatment cohort.

**Figure 8:**
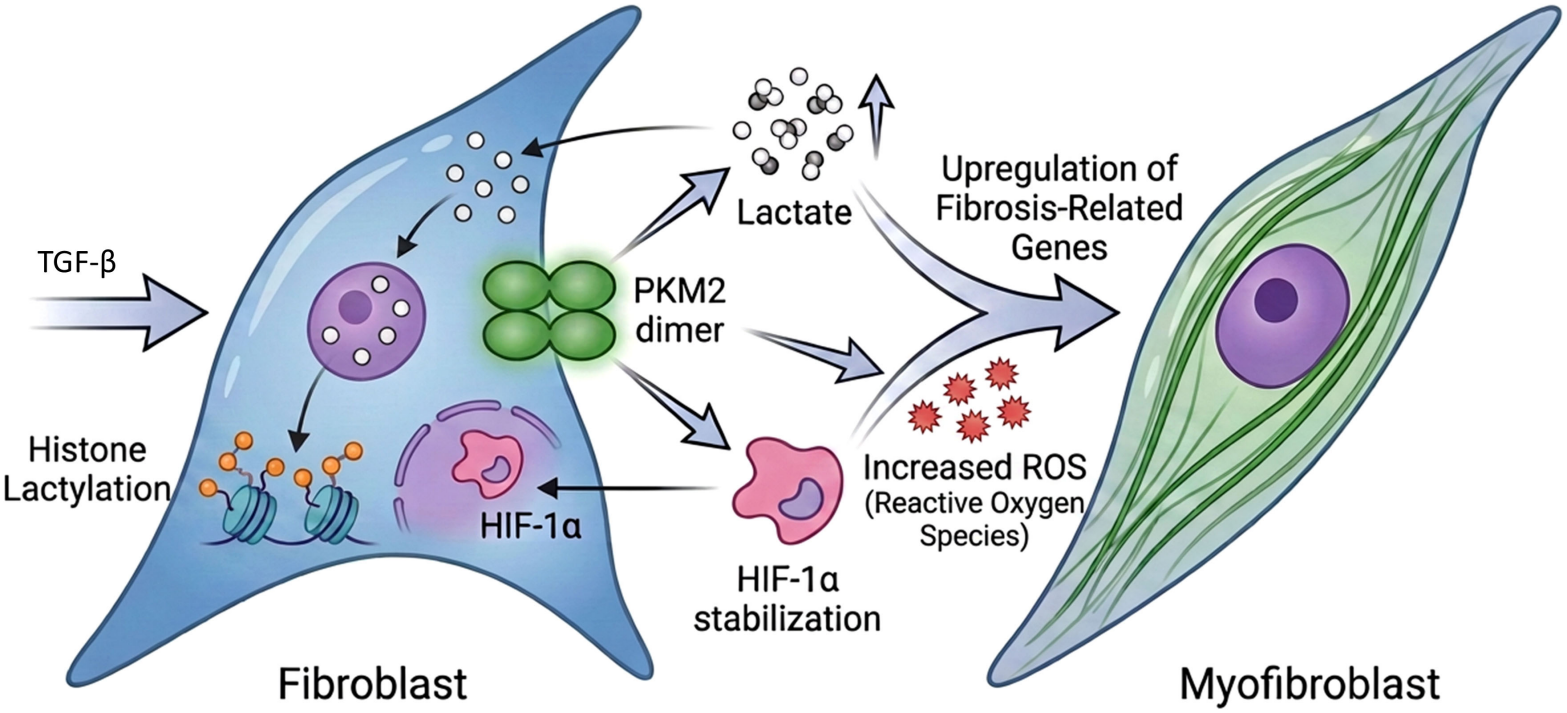
PKM2 governs myofibroblast transition via increased metabolism promoting fibrosis through dual lactylation and HIF-1α stabilisation. Dimeric PKM2 drives glycolysis, leading to elevated lactate, which lactylates histones in the nucleus on specific promoters, thereby facilitating fibrogenic gene expression. Lactate also promotes the stabilisation of HIF-1α via elevated ROS, resulting in enhanced fibrosis-related genes. Forced tetramerisation of PKM2 with TEPP-46 reduced fibrosis, as did inhibition of HIF-1α. TGF-β, transforming growth factor-β.

We present a model in which PKM2 drives a glycolytic switch that promotes myofibroblast transition via an increased glycolytic lactate axis that mediates its effects through HIF-1α and epigenetic alterations (**Figure 8**). Activating PKM2 tetramerisation with TEPP-46 could be a therapeutic strategy in SSc, but this requires further investigation. Such a mechanism, as opposed to global glycolysis inhibition, may yield fewer side effects and less toxicity.

## Data Availability

The raw data supporting the conclusions of this article will be made available by the authors, without undue reservation.
